# LipidXplorer: A Software for Consensual Cross-Platform Lipidomics

**DOI:** 10.1371/journal.pone.0029851

**Published:** 2012-01-17

**Authors:** Ronny Herzog, Kai Schuhmann, Dominik Schwudke, Julio L. Sampaio, Stefan R. Bornstein, Michael Schroeder, Andrej Shevchenko

**Affiliations:** 1 Max Planck Institute of Molecular Cell Biology and Genetics, Dresden, Germany; 2 Department of Internal Medicine III, Carl Gustav Carus Clinics of Dresden University of Technology, Dresden, Germany; 3 National Centre for Biological Sciences, Tata Institute of Fundamental Research, Bangalore, India; 4 Biotechnology Centre, Dresden University of Technology, Dresden, Germany; University College Dublin, Ireland

## Abstract

LipidXplorer is the open source software that supports the quantitative characterization of complex lipidomes by interpreting large datasets of shotgun mass spectra. LipidXplorer processes spectra acquired on any type of tandem mass spectrometers; it identifies and quantifies molecular species of any ionizable lipid class by considering any known or assumed molecular fragmentation pathway independently of any resource of reference mass spectra. It also supports any shotgun profiling routine, from high throughput top-down screening for molecular diagnostic and biomarker discovery to the targeted absolute quantification of low abundant lipid species. Full documentation on installation and operation of LipidXplorer, including tutorial, collection of spectra interpretation scripts, FAQ and user forum are available through the wiki site at: https://wiki.mpi-cbg.de/wiki/lipidx/index.php/Main_Page.

## Introduction

Lipidomics, an emerging branch of *omics* sciences, aims at quantifying all lipid molecules produced by cells, tissues or organisms (reviewed in [1]–[3]). Lipidomics is heavily reliant on mass spectrometry: individual molecular species are identified by their accurate intact masses and/or by masses of specific structural fragments obtained by tandem mass spectrometry (MS/MS) of individual precursors (reviewed in [4]). A palette of biochemical and mass spectrometric methods has been developed to characterize complete lipidomes [5]–[7].

Despite considerable efforts, the concordance between the lipidome composition and quantities of individual species obtained by different analytical approaches from similar samples remains poor. Let us consider, for example, the composition of triacylglycerols (TAG) in human blood plasma – one of the most abundant and easily detectable lipid class in one of the best characterized body fluids. Conflicting numbers of TAG species were reported: 31 [8]; 18 [9] and 24 [10] with significantly different molecular compositions. Graessler *et al* and Oresic *et al* reported, respectively, 6 and 4 species comprising fatty acid moieties with odd numbers of carbon atoms, while Quehenberger *et al* reported none. Quehenberger *et al* pointed to TAG 56∶6 and Graessler *et al* to TAG 56∶8 as the group of isobaric molecules with the highest number of carbon atoms and double bonds, while Pietilainen *et al* [11] identified considerably larger species with 58 carbon atoms and up to 9 double bonds.

Although in population-wide studies the sample origin is an important factor, we argue that automated interpretation of spectra by software remains one of the major sources of lipidome compositional discordances [12]. Consider that lipids constitute a structurally diverse class of biomolecules that are differently ionized and fragmented in MS and MS/MS experiments. Major cleavage pathways of lipid molecular ions are often instrument-dependent (reviewed in [4], [13], [14]). Also, depending on the mass spectrometer design, lipid ions are detected with different mass resolution and accuracy. Therefore, the spectra interpretation software is usually tailored for a certain type of the mass spectrometer and/or spectra acquisition method; it may be underperforming if applied to spectra acquired at alternative instrument platform(s). Additionally, the software usually targets a selection of lipid classes that reflects scientific interests of the developer's team and may not recognize uncommon lipid classes or species - for example, those comprising some unconventional fatty acid moieties, or may be performing sub-optimally.

We also argue that efforts to develop software relying on a single “gold standard” interpretation algorithm may be counterproductive – unless users are able to incorporate their own interpretation algorithms into the same software framework. Along these lines, we developed the software LipidXplorer [12]. LipidXplorer implements the molecular fragmentation query language (MFQL) that can describe any user-defined fragmentation pathway of any lipid class and implement it into a customized spectra interpretation method. LipidXplorer design supports any shotgun lipidomics routine, from high throughout top-down screening for molecular diagnostics and biomarker discovery to the targeted absolute quantification of low abundant lipid species [6], [8], [15]–[20].

Here we provide evidence that the LipidXplorer software also enables qualitatively and quantitatively consistent interpretation of lipid spectra acquired by different methods on different mass spectrometers independently of any resource of reference mass spectra.

## Design and Implementation

LipidXplorer is programmed in Python 2.6 and has a modular architecture [12] that supports the identification and quantification of lipids in large collections of shotgun (*i.e.* acquired by direct infusion of total lipid extracts, reviewed in [1]) MS and MS/MS spectra. Basically, the software imports raw spectra in the generic file formats mzXML [21] or .csv/.dta (peak lists), considering common peak attributes such as mass accuracy, mass resolution and the slope of its change along with increasing *m/z*. Individual MS and MS/MS spectra are usually acquired within some period of time as a series of discrete scans. LipidXplorer first merges scans into representative spectra. Next, it aligns individual peaks in related MS and MS/MS spectra acquired from different samples and, within each cluster of aligned peaks, substitutes their masses with the single intensity weighted average mass, while their abundances in each individual acquisition are preserved. Representative masses of aligned peak clusters and individual peak intensities are stored in a flat-file database (MasterScan) composed using the “pickle” module (http://docs.python.org/library/pickle.html) for object serialization of the Python standard library.

While processing spectra for the MasterScan, LipidXplorer imposes two experiment-specific constraints. The first constraint defines the minimal intensity of precursor and fragments peaks. The second constraint, termed the occupancy threshold, is related to the frequency at which a particular peak was observed in all acquired spectra; for example, setting the threshold to 100% implies that the MasterScan will only encompass peaks observed in each and every spectrum. These constraints help to adjust processing settings to the actual level of chemical noise and harmonize the confidence of lipid species identification between independent experiments. We underscore that spectra are processed independently of a mass spectrometer type: they are imported in generic machine-independent formats and, while building a MasterScan, only peak attributes reflecting basic instrument- dependent spectra features, are considered.

**Figure 1:**
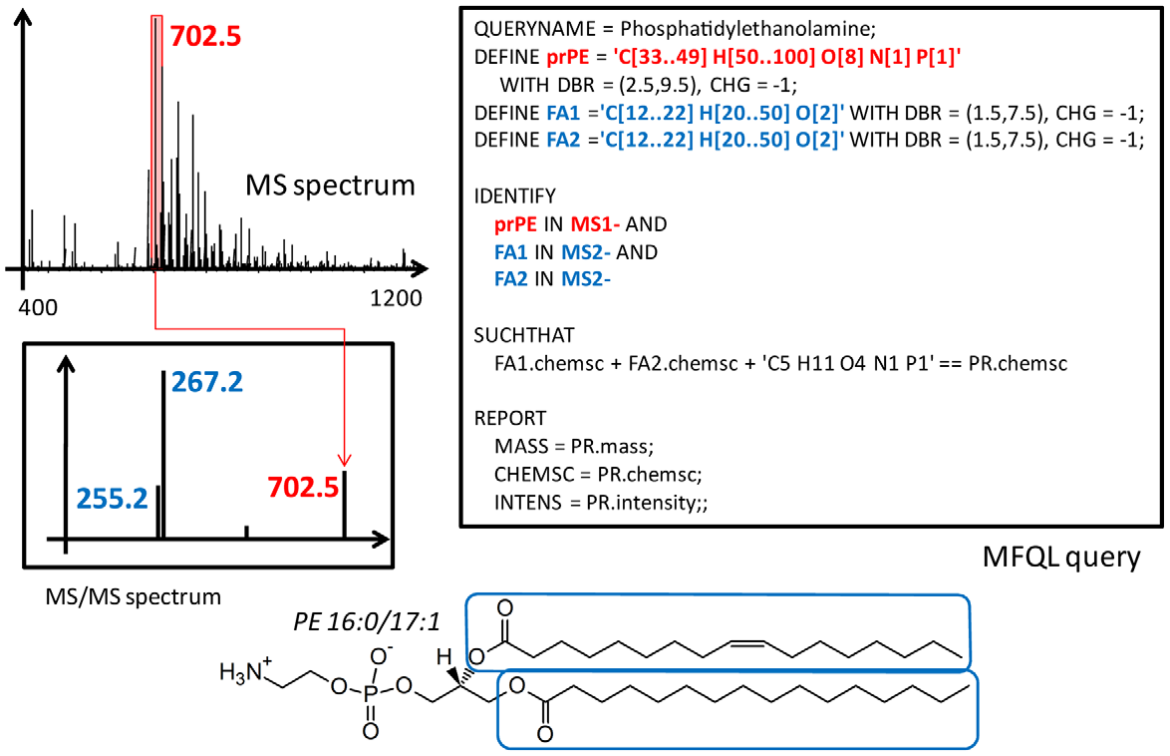
MFQL query for identifying phosphatidylethanolamine (PE) species in MS/MS spectra. PE molecules consist of a glycerol backbone to which the phosphoethanolamine head group and two fatty acid moieties are attached *via* phosphoether and ester bonds, respectively. The chemical structure of PE 16∶0/17∶1 in its zwitterion form is shown at the bottom panel with two fatty acid moieties (16∶0 and 17∶1) boxed; other PE species may differ by their fatty acid moieties. If identified by precursor *m/z* and/or lipid-class specific fragment (for example, originating from the phosphoethanolamine head group), lipids are annotated by their class and total number of carbon atoms and double bonds in both fatty acid moieties (in this case, PE 33∶1). However, by identifying both fatty moieties the analysis may recognize the individual molecular species (here PE 16∶0/17∶1). Usually the location of fatty acid moieties at the glycerol backbone (*sn-*1 or *sn-*2) could be inferred from the relative abundance of corresponding acyl anions. In this example, PE molecular anion having *m/z* 702.5 was detected in the survey MS spectrum (here termed as MS1-) and then its MS/MS spectrum (here termed as MS2-) was acquired. The latter was dominated by abundant acyl anion fragments (*m/z* 255.2 and 267.2) originating from 16∶0 and 17∶1 fatty acid moieties, respectively. Other species of PE class will fragment similarly. During the shotgun experiment all peaks of plausible PE precursors will be fragmented. To identify PE species, we first DEFINE the sum composition constraints (rather than exact values of expected masses) for intact PE molecules (prPE) and acyl anion fragments (FA1 and FA2); we also expect them to be singly charged (CHG = −1) and that their unsaturation (expressed as the double bond equivalent range, DBR) should be within 1.5 to 7.5. The next section requests to IDENTIFY previously DEFINE(d) precursors in MS1- spectrum and fragments in corresponding MS2- spectra SUCHTHAT sum compositions of both fatty acid moieties (FA1.chemsc and FA2.chemsc), together with the phosphoethanolamine head group and glycerol backbone (C5 H11 O4 N1 P1), add up to the sum composition of the intact precursor (PR.chemsc). Next, the ‘REPORT’ section describes the data output format. Here the query requests to report the masses (MASS), sum compositions (CHEMSC) and intensities (INTENS) of all matched PE precursors (prPE.intensity); more elaborate queries may also name the identified species according to a user-defined convention and report intensities of relevant fragment ions along with corresponding mass measurement errors. Further details on MFQL format and syntax are provided at the LipidXplorer wiki site at: https://wiki.mpi-cbg.de/wiki/lipidx/index.php/LipidXplorer_MFQL.

The MasterScan database holds all MS and MS/MS spectra acquired from all samples in the experiment and can be further interrogated by queries written in the molecular fragmentation query language (MFQL). The MFQL interpreter is written using PLY (Python Lex-Yacc) (http://www.dabeaz.com/ply/), a lexer/parser generator based on Lex and Yacc. Basically, each query defines what structure-specific “signature” ions and/or their *boolean* combinations should be recognized in MS and MS/MS spectra within the MasterScan. Both precursor and fragment ions could serve as “signatures” leading to unequivocal identification of species in complex lipid extracts [12], [22] (Figure 1). Typically, each query targets one lipid class, while many queries can be successively executed by the LipidXplorer. Lipid species identified by all executed queries are reported in a single results file. A collection of basic MFQL scripts covering major lipid classes is included into the distributed version of LipidXplorer and is available at its wiki site: https://wiki.mpi-cbg.de/wiki/lipidx/index.php/Main_Page.

The MFQL query also defines the format in which the identified species are reported: the results file in .csv format provides the list of identified lipid species annotated according to user-defined rules and the abundances of corresponding fragment and/or precursor ions for the subsequent quantification of species.

## Results

We previously demonstrated that, because of the two conceptually novel solutions – MasterScan and MFQL, LipidXplorer enabled accurate interpretation of shotgun lipidomics datasets acquired on different tandem mass spectrometers [12]. One factor contributing to the interpretation consistency was that, irrespective of the employed instrument, we processed similarly structured datasets obtained by data-dependent acquisition. They consisted of survey MS spectra and full MS/MS spectra acquired either from peaks detected in survey spectra, or from peaks whose masses matched the masses from a pre-compiled inclusion list. Although data-dependent acquisition is a powerful approach [20], [22]–[24] it is only applied on rapid scanning high mass resolution tandem mass spectrometers, such as hybrid quadrupole time-of-flight (reviewed in [25]) or LTQ Orbitrap [26], [27]. However, currently the largest body of shotgun lipidomics work is performed using triple quadrupole or triple quadrupole - linear ion trap (QTRAP) mass spectrometers (reviewed in [1], [4], [14], [28], [29]) by precursor- or neutral loss scanning [9], [30]. In these analyses no full MS/MS spectra are acquired: the instrument is set to detect one particular fragment (precursor ion scanning), or fragments with pre-defined mass difference to the fragmented precursor (neutral loss scanning) that are originating from all precursors within certain *m/z* range. In each analysis, only one fragment mass (in case of precursor ion scanning) or mass difference (in case of neutral loss scanning) is monitored and then the analysis is repeated for the next chosen fragment mass/mass difference. These analyses produce differently structured datasets to which generic “full MS/MS spectra” interpretations are not applicable directly.

**Figure 2:**
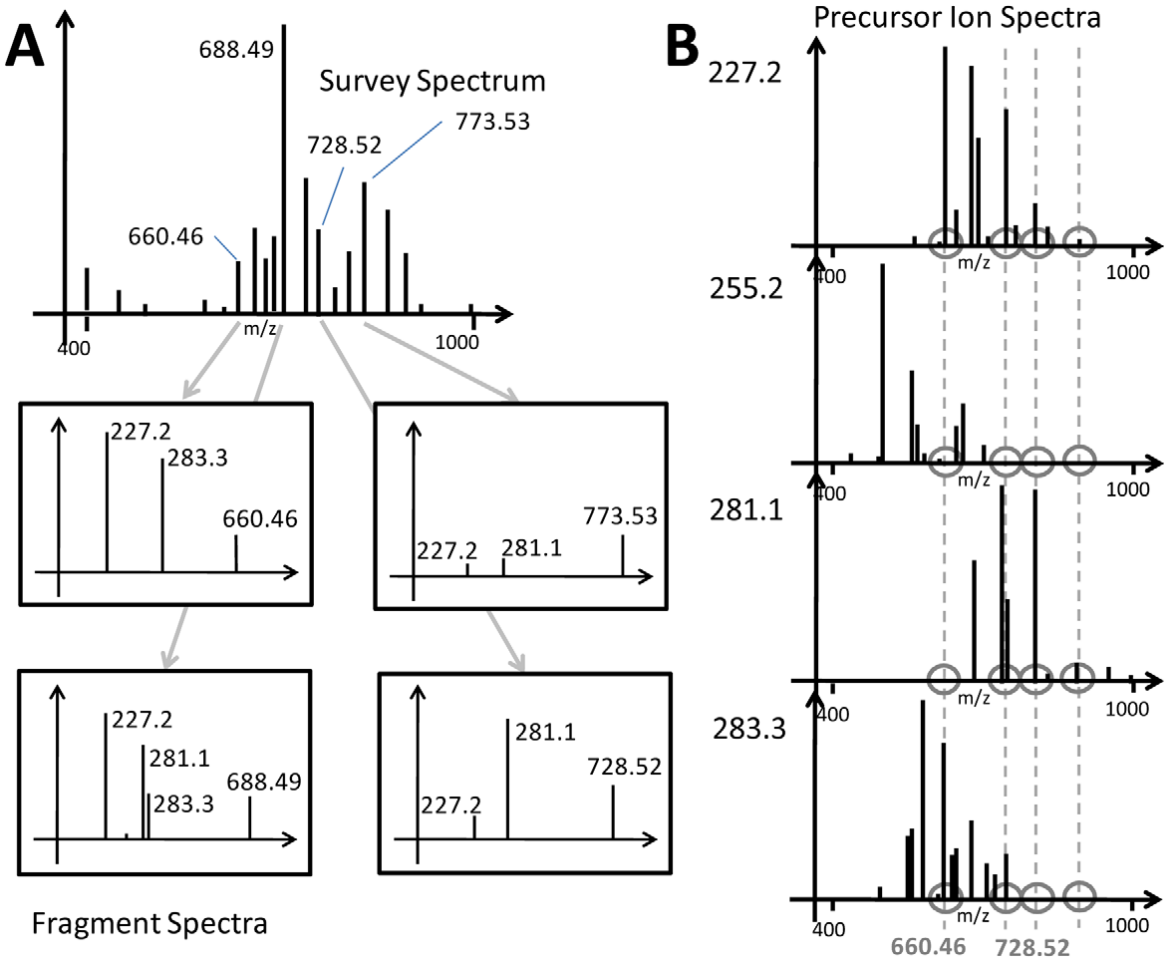
DDA-driven MS/MS and Precursor Ion Scanning (PIS) Spectra. The scheme explains how data-dependent acquisition of full MS/MS spectra (DDA-driven MS/MS) and precursor ion scanning spectra are related. In DDA mode (panel A) a tandem mass spectrometer first acquires a survey spectrum that determines masses of intact lipids (here we are showing precursors with *m/z* 660.46; 688.49; 728.52 and 773.53 as an example) and then acquires full MS/MS spectra (from *m/z* of a lowest expected fragment till *m/z* of the intact precursor) from all plausible precursors. In MS/MS spectra (panel A) we designated *m/z* of characteristic acyl anion fragments (*m/z* 227.2; 281.1; 283.3) produced from fatty acid moieties of molecular anions of glycerophospholipids. In PIS spectra (panel B) a mass spectrometer registers the intensity of one pre-selected fragment (in this example, one of the acyl anion fragments) produced from all precursor masses within the specified *m/z* range. Hence, only precursors yielding the specific fragment will produce a peak, while others will not. Usually, on triple quadrupole mass spectrometers PIS spectra for a large number of fragments (like, all acyl anions of all major fatty acids) are acquired successively. Subsequent alignment of PIS spectra reveals what expected fragments were produced from each precursor (dotted line). For example, a lipid with *m/z* 660.46 produced acyl anions with *m/z* 227.2 and 283.3 that correspond to 14∶0 and 18∶0 fatty acids. The scheme exemplifies that DDA-driven MS/MS and PIS produce complementary structural evidence, although they originate from two completely different modes of spectra acquisition.

Here we demonstrate how low mass resolution precursor ion spectra and neutral loss spectra can be interpreted by LipidXplorer and provide evidence that these interpretations are consistent with alternative analyses by DDA-driven acquisition of full MS/MS spectra. Let us explain the interpretation algorithm using a precursor ion scan spectrum as an example. A precursor ion scanning spectrum plots the abundance of one specific fragment ion produced from a precursor isolated within the resolution-dependent mass window that is moving with a certain small increment, usually 0.05 to 0.2 Th, along *m/z* range (Figure 2). If several precursor ion spectra are acquired, they can be aligned by precursor masses and then transposed to the format: [precursor mass]: [frag1, abundance], [frag2, abundance], …, [frag*n*, abundance], where the precursor masses are all masses within the considered mass range. This operation corresponds to the transformation of spectra in panel B to spectra in panel A, however the latter only comprises a restricted set of fragment masses. The spectra alignment algorithm employed by LipidXplorer [12] effectively creates a “virtual” MS/MS spectrum for each precursor mass observed in individual precursor ion spectra: the only difference with shotgun datasets acquired by DDA is that they comprise no survey MS spectra. The algorithm is also adjustable to the actual mass resolution and accuracy. Therefore, upon building a MasterScan from transposed spectra, lipid identification could proceed with the same MFQL queries in the usual way.

**Figure 3:**
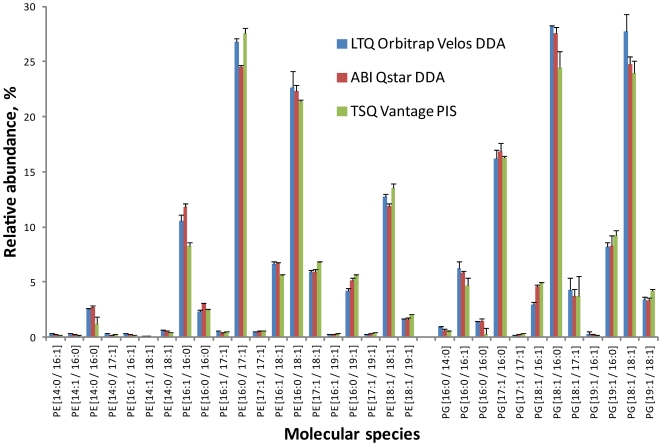
Comparison of the lipid profiles obtained by three independent analytical methods. Total lipid extract from *E.coli* was analyzed on the QSTAR and LTQ Orbitrap Velos mass spectrometers in DDA mode and on the TSQ Vantage triple quadrupole mass spectrometer by precursor ion scanning for acyl anion fragments. The same MFQL queries were employed to identify and quantify lipids of PE and PG classes. Cardiolipins, another major component of the *E.coli* lipidome, were omitted from the comparative test because their precursors were detected in two charge states and the interpretation might be biased by the instrument interface settings and mass resolution. Relative abundances of individual species were normalized to the total abundance of all species of each class. Error bars represent standard deviations (SD, n = 3 for experiments on the TSQ Vantage and n = 4 on the QSTAR and LTQ Orbitrap mass spectrometers). Relative abundances determined on LTQ Orbitrap and QSTAR correlated with r2 and slope of 0.99 and 0.94, respectively; on LTQ Orbitrap and TSQ Vantage: r2 = 0.98 and slope 0.93; QSTAR and TSQ Vantage r2 = 0.98 and slope 0.98.

To validate cross-platform interpretation capabilities of LipidXplorer, we analyzed a commercial sample of the total lipid extract from *E.coli* (Avanti Polar Lipids, Alabaster, AL) by shotgun experiments performed in different analytical modes and on different mass spectrometers. Upon collision induced fragmentation, molecular anions of phosphatidylethanolamines (PE) and phosphatidylglycerols (PG) – the major constituents of *E.coli* lipidome produce abundant acyl anion fragments of their fatty acid moieties that, together with precursor masses, unequivocally identify their molecular species [5], [29], [31], [32] (Figure 1). We first acquired MS/MS spectra from all PE and PG precursors on a quadrupole time-of-flight instrument QSTAR Pulsar *i* and on LTQ Orbitrap Velos using data-dependent acquisition in negative ion mode. Molecular anions were selected with the unit mass resolution to prevent co-fragmenting neighboring precursors. Collision energies were optimized as described in [20], [32] and, in the course of analyses, either ramped with precursor masses within the range of 44 to 56.5 eV (*m/z* 600 to 850) (QSTAR) or applied as a normalized collision energy nCE = 45% [20] (LTQ Orbitrap Velos). For better consistency, spectra were acquired with approximately the same mass resolution of 7 500 (full width at half maximum, FWHM) for both LTQ Orbitrap Velos and QSTAR; the impact of mass resolution on lipid identification accuracy was examined in [12]. Experiments were performed in 4 replicas; a shotgun dataset comprising 31 MS and 321 MS/MS spectra was processed and individual species were quantified (Figure 3). Note that *E.coli* does not produce ether glycerophospholipids. If analyzed by precursor scanning for acyl anions of fatty acid moieties, identification of ether lipids would rely on matching a single acyl anion to the precursor mass since complementary alkoxide fragment is usually *ca* 20-fold less abundant [32]. In positive mode plasmenyl species of PE could be distinguished from plasmanyl species by specific fragments [33] accountable *via boolean* scans [23].

In parallel, the same extract was infused into a triple quadrupole mass spectrometer TSQ Vantage (Thermo Fisher Scientific) and 72 precursor ion scan spectra were successively acquired for masses of acyl anion fragments of common fatty acids, including all fatty acids recognized in the experiment above. Spectra were also acquired under unit mass resolution consistently with the experiment settings applied on the QSTAR and LTQ Orbitrap; collision energy (CE) was 50 eV; collision gas pressure 1.5 mTorr. The MasterScan was composed from the aligned and transformed spectra and interpreted by the same MFQL queries identifying species of PE and PG lipid classes [12]. To quantify the species, isotopic correction of precursors and fragment intensities was applied [12], [20]. We found that quantitative profiles obtained in three independent experiments on hybrid tandem machines QSTAR and LTQ Orbitrap and on a triple quadrupole Vantage instrument, were consistent (Figure 3).

**Figure 4:**
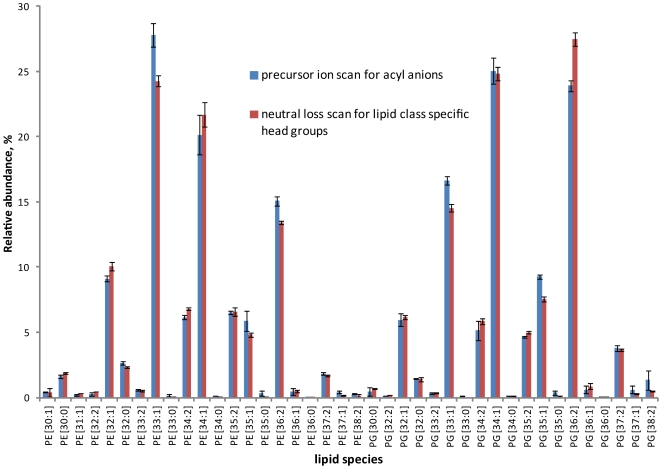
Comparison of the lipid profiles obtained by precursor ion scanning and neutral loss scanning on a triple quadrupole mass spectrometer. Total lipid extract from *E.coli* was analyzed in negative mode on the TSQ Vantage triple quadrupole mass spectrometer by precursor ion scanning for acyl anion fragments (profiles are the same as in Figure 3). The same extract was analyzed in positive mode by lipid-class specific neutral loss scanning for the loss of head groups of PE and PG: Δ *m/z* 141.02 for [M+H]^+^ molecular ions of PE and Δ *m/z* 189.04 for ammonium adducts [M+NH_4_]^+^ of PG. Relative abundances of individual species were normalized to the total abundance of all species of each class. Error bars represent standard deviations (SD, n = 3 for experiments on the TSQ Vantage). Relative abundances of species determined on TSQ Vantage by precursor ion scanning and neutral loss scanning correlated with r2 and slope of 0.98 and 0.94 for PE and r2 = 0.98 and slope 1.03 for PG.

Using the same *E.coli* lipid extract, we further tested if LipidXplorer could consistently interpret neutral loss scanning spectra. Upon collisional fragmentation in positive ion mode, molecular cations of PE and ammonium adducts of PG undergo facile neutral losses of their head groups (Δ *m/z* 141.02 and Δ *m/z* 189.04, respectively), which are conventionally used for their shotgun profiling [23], [34]. We performed shotgun neutral loss experiments on the TSQ Vantage under basic instrument settings similar to described above; however spectra were acquired in positive mode under CE = 22 eV. Spectra were acquired in three replicas, processed using MFQL queries accounting for the head group neutral losses (available at the LipidXplorer wiki site) and normalized abundances of species compared (Figure 4). Note that in neutral loss scanning spectra identified lipids may only be annotated by the total number of carbons and double bonds in both fatty acid moieties. For consistent comparison with profiles deduced from negative mode precursor ion spectra (Figure 3), the abundances of isobaric species of the same lipid class were combined. For each lipid class, we observed good correlation between the profiles, suggesting that LipidXplorer consistently interpreted both precursor ion scanning and neutral loss scanning spectra.

These experiments demonstrated that LipidXplorer informatics concept is generic and offers consistent interpretation of shotgun datasets irrespectively of the instrument platform and acquisition mode. Hence, it enables direct quantitative comparison of lipid species profiles acquired from complex lipid extracts in different laboratories by any shotgun methodology and constitutes an important step towards consensual instrument platform-independent lipidomics.

## Availability and Future Directions

Full documentation on the LipidXplorer, lipid identification tutorial, library of MFQL scripts and sample spectra datasets are provided at its wiki page: https://wiki.mpi-cbg.de/wiki/lipidx/index.php/Main_Page. LipidXplorer is licensed under the GPL and freely distributed from: https://sourceforge.net/projects/lipidxplorer/files/. It is written in Python 2.6 and is directly provided as a source code. The installer and installation guidelines are at: https://wiki.mpi-cbg.de/wiki/lipidx/index.php/LipidXplorer_Installation.

We demonstrated that, because of its innovative design concept, LipidXplorer consistently interprets shotgun spectra acquired in any mode (MS, data-dependent MS/MS, precursor and neutral loss scanning) by any tandem mass spectrometer (quadrupole time-of-flight, linear ion trap Orbitrap or triple quadrupole). LipidXplorer imports and aligns MS and MS/MS spectra and identifies lipids under resolution, noise thresholds, tolerance and identification settings that can be customized for any mass spectrometer, acquisition mode or dataset. Since spectra interpretation does not rely on a reference database, the software could encompass species of any lipid class that were ionized and fragmented during a shotgun experiment. The same MFQL-based approach could be applied to identify lipids in LC-MS/MS spectra. However, species quantification would require a separate module for time-integrating their chromatographic peaks.

While it has become possible to consistently interpret shotgun spectra, the question remains: how accurate are those identifications for a given dataset and experiment settings? No statistical framework has yet been developed to either estimate a global false discovery rate, or compute the local probability that a particular assignment is correct. In some instances it should be possible to revert to manual inspection of acquired spectra, yet this approach is biased and hardly applicable to large scale lipidomics efforts.

It is also important to combine lipid identifications with project-dependent data processing and visualization tools for integrating quantitative lipidomic profiles into the specific context of on-going efforts in cell biology and molecular medicine.
